# Supplementary material for: The relationship between smoking cigarettes and metabolic syndrome: A cross-sectional study with non-single residents of Seoul under 40 years old
Source: PLoS One. 2021 Aug 19;16(8):e0256257. doi: 10.1371/journal.pone.0256257 (PMC8376018; doi:10.1371/journal.pone.0256257)
Supplement: Attachment — Submitted filename: PONE-D-20-15624_R1.pdf [file pone.0256257.s004.pdf]

PLOS ONE

The relationship between smoking cigarettes and metabolic syndrome: A cross-sectional study with non-single residents of Seoul under 40 years old

--Manuscript Draft--

Manuscript Number:	PONE-D-20-15624R1
Article Type:	Research Article
Full Title:	The relationship between smoking cigarettes and metabolic syndrome: A cross-sectional study with non-single residents of Seoul under 40 years old
Short Title:	Relationship between smoking and metabolic syndrome among urban non-single young populations.
Corresponding Author:	Bumjo Oh, M.D.<br>Seoul National University Seoul Metropolitan Government Boramae Medical Center<br>Seoul, KOREA, REPUBLIC OF
Keywords:	non-single residents; health screening; cigarette smokers; Metabolic Syndrome
Abstract:	Introduction: Young adults receive health screenings at lower rates than other age groups, and it may be difficult to detect diseases in the early stages for this group. We examined differences in health status relative to smoking in a young age group using the results of health screenings conducted in engaged and newly married couples in a cross-sectional database.<br>Methods: The participants in this study were 808 young adults who visited a municipal hospital health screening center from July 2017 to March 2019. They completed a self-administered questionnaire, and physical measurements and a blood test were taken. They were classified into non-cigarette smokers, past cigarette smokers, and current cigarette smokers according to smoking behavior. In this study, we compared metabolic syndrome, the main components of which include obesity, high blood pressure, high blood triglycerides, low levels of HDL cholesterol and insulin resistance, with smoking behavior.<br>Results: The mean age of the participants was 30.9±3.3 years (males 32.0±3.2, females 29.8±3.1), and 13.9% were current cigarette smokers (males 22.8%, females 5.1%). The proportion of men in their 30s was 76.6% for male group and 50.0% for female group, indicating that the male group had a relatively higher proportion of older and current smokers. Significant differences were found in age, sex, blood pressure, metabolic abnormalities, and drinking status according to smoking status. Cigarette smokers had a 2.4-fold greater risk of metabolic syndrome (95% confidence interval [CI], 1.43–3.96) than non-cigarette smokers; in particular, they had a 2.6-fold (95% CI, 1.44–4.55) greater risk of hypertriglyceridemia and a three-fold (95% CI, 1.45–6.35) greater risk of low HDL cholesterol.<br>Conclusions: In comparison with non-single, young and generally healthy city dwellers, the risk of metabolic syndrome was significantly higher in smokers than in non-smokers, and in particular, it was confirmed that the risk of hypertriglyceridemia and low HDL cholesterolemia was higher. Smoking cessation is necessary, even for the young, because smoking may cause changes in blood lipids even if the smoking duration is short.
Order of Authors:	Sun Woo Kim
	Ho Jun Kim
	Kyungha Min
	Hobeom Lee
	Sung-Ha Lee
	Sunyoung Kim
	Jong Seung Kim
	Bumjo Oh, M.D.
Response to Reviewers:	Dear Editor,

Powered by Editorial Manager® and ProduXion Manager® from Aries Systems Corporation

We like to thank you and the reviewers for the very helpful advices.
Please find our revision and answers to the open points enclosed.

Best regards,

ABSTRACT

1)Title – I am not quite sure why the authors have chosen 'marriage' as a determinant of metabolic syndrome? Marriage may not happen, and people can live together for decades. It does not affect their risk of developing metabolic syndrome.
Answer: We agree with the reviewer's opinion. This was a kind of business, and the purpose was to recommend the health care of newlyweds, and I think that the title of this manuscript, written for research purposes, needs to be revised. So, we have changed it to:
The relationship between smoking cigarettes and metabolic syndrome: a cross-sectional study with non-single residents of Seoul under 40 years old

2)Metabolic syndrome was defined using Adult Treatment Panel III.- Please tell the reader what is meant by the adult treatment panel.
Answer: Thank you for your comment. The content was explained by quoting from the methodology, not the abstract.

3)Results – Please add standard deviation of age and male female split. This is going to help the reader to understand dispersion of data. Male female split will be helpful as you have mentioned the uptake of screening tests are comparatively lower in young males.
Answer: Thank you for your comment. Men and women were separated and SD was indicated.

4)Conclusion – Glycosylated Haemoglobin, smoking and metabolic syndrome are closely linked. Why the authors only commented on TG and HDL and not other components of the metabolic syndrome?
Answer: In this study, we looked at the relationship between smoking and metabolic syndrome, and further looked at the relationship between the components that define metabolic syndrome. Through this, the Conclusion emphasized and mentioned this part with the result that TG and HDL had a significant relationship with smoking.

INTRODUCTION

1)The annual number of marriages in South Korea showed increases over a long period – correct grammar.
Answer: We have corrected the sentence as you pointed out.

2)"Metabolic syndrome is the composite occurrence of abdominal obesity, dyslipidemia, elevated blood pressure, and impaired fasting glucose" – what about BMI and waist circumference?
Answer: Thank you for your comment. The relationship between obesity index and metabolic syndrome risk factors has been suggested in various ways, and the authors selected one of them according to the criteria of Adult Treatment Panel III. This is because BMI and waist circumference are both used as indicators of obesity, but if they are selected at the same time, covariance may play a role.

3)"Therefore, this study is intended to conduct cross-sectional studies based on the results of health screenings of engaged and newly married couples who visited the health examination center of a general hospital in Seoul from July 2017 to March 2019 in order to analyze differences in the health status, various clinical tests, and prevalence between smoking and non-smoking among existing living habits in relatively young age groups" – too long sentence not sure what the authors are trying to say. Please rewrite the sentence.
Answer: We agree with the reviewer's opinion, and have corrected the sentence as pointed out.

Powered by Editorial Manager® and ProduXion Manager® from Aries Systems Corporation

STUDY METHOD
1)Statistical analysis – “In addition, after compensating for age, sex, drinking, ……” adjusting would be a better word than compensating.
Answer: We agree with the reviewer's opinion, and changed the word as you instructed.

2)Table 3 – Can the author clarify what is meant by crude OR and adjusted OR, please?
Answer: As you have pointed out, we added a description in Table 3.

DISCUSSION
1)Why the authors decided only to comment of lipid profiles rather than the whole spectrum of components of metabolic syndrome?
Answer: As mentioned in the Conclusion, this study confirmed that the prevalence of metabolic syndrome was higher in the current smoker group than in the non-smoker group. In particular, it was confirmed that the adjusted OR significantly increased in TG and HDL-C (high-density lipoprotein) among metabolic components. In this regard, the discussion was focused on the relevant risk factors. The authors emphasize this intention by amending the first sentence of the first paragraph of the Discussion.

2)Relationship between smoking and the components of metabolic syndrome is well established - https://pubmed.ncbi.nlm.nih.gov/27881170/
Answer: Thank you for your comment. We cited and mentioned it in the last sentence of the second paragraph of the Discussion after reviewing the contents of the review article.

3)The authors could inform the reader how this relationship is different in young people in their 20s and 30s.
Answer: Thank you for your comment. The aforementioned papers are included as references. In addition, compared with previous studies, a significant increase in TG and a significant decrease in HDL were characteristic in smokers who were younger subjects under the age of 40, and this was mentioned in the Discussion section.

5. Review Comments to the Author
Please use the space provided to explain your answers to the questions above. You may also include additional comments for the author, including concerns about dual publication, research ethics, or publication ethics. (Please upload your review as an attachment if it exceeds 20,000 characters)
Reviewer #1: This is an important area of research to explore how smoking affects the cardiometabolic parameters in people in their 20s and 30s. This paper can be published with some minor corrections. The paper needs overall editing for grammar and punctuation.
Answer: Thank you for your comment. We have completed language editing once again for the revised manuscript.

Reviewer #2: Obesity and smoking are important causes of morbidity and mortality worldwide. Smoking reduces insulin sensitivity, induces insulin resistance, and enhances cardiovascular risk factors, such as elevated plasma triglycerides, reduced high-density lipoprotein–cholesterol, and hyperglycemia. Smoking is associated with metabolic abnormalities and increases the risk of metabolic syndrome.
In this study, the authors evaluated the relationship between smoking and metabolic syndrome in 808 young adults and found that the odds ratio versus nonsmokers for metabolic syndrome, hypertriglyceridemia, and low HDL cholesterolemia was significantly higher in smokers. This study provides a positive association between smoking and metabolic syndrome in young adults.
Some questions need to be addressed. Whether and how drinking affects metabolic syndrome in young adults either in the smoking group or non-smoking group. Was any difference of metabolic syndrome analyzed in the light smokers and heavy smokers, compared to the non-smoking group?
Answer: Thank you for your comments, and we would like to respond to them.
First, the researchers divided the subjects into three groups, namely current smokers,

	non-smokers, and ex-smokers, according to the definition of ever-smoker, and analyzed them.<br>1) Since the number of subjects is not large, if the categories are further divided by the new definition, the statistical power may decrease.<br>2) The definition of light/heavy smoker is not clear.<br>3) The smoking rate of female subjects is very low.<br>For these reasons, the authors judged that it would be difficult to identify a significant difference between groups when additional conditions were added for analysis.<br>However, we thought that there is a possibility of a dose-response between the amount of smoking and metabolic syndrome, so this was additionally described in the Discussion section.<br>Second, we are well aware that alcohol consumption is also a major contributor to metabolic syndrome. However, it was later realized that the authors did not accurately determine the type and amount of alcohol the subjects drank when collecting the questionnaire. For convenience, since "if you drink more than once a week" is defined as a drinker, it is acknowledged that the proportion of drinkers is high. Therefore, the authors agreed that a more meaningful result would have been possible if the relationship with metabolic components was further checked by dividing it into appropriate/risk drinking according to the amount of alcohol consumed. Reflecting these details, an existing reference was added and this content was additionally described as a limitation in the Discussion section.
**Additional Information:**	
**Question**	**Response**
**Financial Disclosure**<br><br>Enter a financial disclosure statement that describes the sources of funding for the work included in this submission. Review the submission guidelines for detailed requirements. View published research articles from *PLOS ONE* for specific examples.<br><br>This statement is required for submission and **will appear in the published article** if the submission is accepted. Please make sure it is accurate.	No

**Unfunded studies**<br>Enter: *The author(s) received no specific funding for this work.*<br><br>**Funded studies**<br>Enter a statement with the following details:<br>• Initials of the authors who received each award<br>• Grant numbers awarded to each author<br>• The full name of each funder<br>• URL of each funder website<br>• Did the sponsors or funders play any role in the study design, data collection and analysis, decision to publish, or preparation of the manuscript?<br>• **NO** - Include this sentence at the end of your statement: *The funders had no role in study design, data collection and analysis, decision to publish, or preparation of the manuscript.*<br>• **YES** - Specify the role(s) played.<br><br>* typeset	
**Competing Interests**<br><br>Use the instructions below to enter a competing interest statement for this submission. On behalf of all authors, disclose any competing interests that could be perceived to bias this work—acknowledging all financial support and any other relevant financial or non-financial competing interests.<br><br>This statement **will appear in the published article** if the submission is accepted. Please make sure it is accurate. View published research articles from *PLOS ONE* for specific examples.	The authors have declared that no competing interests exist.

**NO authors have competing interests**<br><br>Enter: *The authors have declared that no competing interests exist.*<br><br>**Authors with competing interests**<br><br>Enter competing interest details beginning with this statement:<br><br>*I have read the journal's policy and the authors of this manuscript have the following competing interests: [insert competing interests here]*<br><br>* typeset	
**Ethics Statement**<br><br>Enter an ethics statement for this submission. This statement is required if the study involved:<br><br>• Human participants<br>• Human specimens or tissue<br>• Vertebrate animals or cephalopods<br>• Vertebrate embryos or tissues<br>• Field research<br><br>Write "N/A" if the submission does not require an ethics statement.<br><br>General guidance is provided below. Consult the submission guidelines for detailed instructions. **Make sure that all information entered here is included in the Methods section of the manuscript.**	This study was approved by the institutional review board of SMG-SNU Boramae Medical Center (registration number:30-2018-101). Written informed consent confirming voluntary participation was obtained from the participants. All individually identifying records were anonymized prior to analysis.

Powered by Editorial Manager® and ProduXion Manager® from Aries Systems Corporation

**Format for specific study types**<br><br>**Human Subject Research (involving human participants and/or tissue)**<br>• Give the name of the institutional review board or ethics committee that approved the study<br>• Include the approval number and/or a statement indicating approval of this research<br>• Indicate the form of consent obtained (written/oral) or the reason that consent was not obtained (e.g. the data were analyzed anonymously)<br><br>**Animal Research (involving vertebrate animals, embryos or tissues)**<br>• Provide the name of the Institutional Animal Care and Use Committee (IACUC) or other relevant ethics board that reviewed the study protocol, and indicate whether they approved this research or granted a formal waiver of ethical approval<br>• Include an approval number if one was obtained<br>• If the study involved *non-human primates*, add *additional details* about animal welfare and steps taken to ameliorate suffering<br>• If anesthesia, euthanasia, or any kind of animal sacrifice is part of the study, include briefly which substances and/or methods were applied<br><br>**Field Research**<br><br>Include the following details if this study involves the collection of plant, animal, or other materials from a natural setting:<br>• Field permit number<br>• Name of the institution or relevant body that granted permission	
**Data Availability**<br><br>Authors are required to make all data underlying the findings described fully available, without restriction, and from the time of publication. PLOS allows rare exceptions to address legal and ethical concerns. See the PLOS Data Policy and FAQ for detailed information.	Yes - all data are fully available without restriction

A Data Availability Statement describing where the data can be found is required at submission. Your answers to this question constitute the Data Availability Statement and **will be published in the article**, if accepted.<br><br>**Important:** Stating 'data available on request from the author' is not sufficient. If your data are only available upon request, select 'No' for the first question and explain your exceptional situation in the text box.<br><br>Do the authors confirm that all data underlying the findings described in their manuscript are fully available without restriction?	
**Describe where the data may be found in full sentences. If you are copying our sample text, replace any instances of XXX with the appropriate details.**<br><br>• If the data are **held or will be held in a public repository**, include URLs, accession numbers or DOIs. If this information will only be available after acceptance, indicate this by ticking the box below. For example: *All XXX files are available from the XXX database (accession number(s) XXX, XXX.).*<br>• If the data are all contained **within the manuscript and/or Supporting Information files**, enter the following: *All relevant data are within the manuscript and its Supporting Information files.*<br>• If neither of these applies but you are able to provide **details of access elsewhere**, with or without limitations, please do so. For example:<br><br>*Data cannot be shared publicly because of [XXX]. Data are available from the XXX Institutional Data Access / Ethics Committee (contact via XXX) for researchers who meet the criteria for access to confidential data.*<br><br>*The data underlying the results presented in the study are available from (include the name of the third party*	All relevant data are within the manuscript and its Supporting Information files.

and contact information or URL).<br>• This text is appropriate if the data are owned by a third party and authors do not have permission to share the data.<br><br>* typeset	
Additional data availability information:	Tick here if your circumstances are not covered by the questions above and you need the journal’s help to make your data available.

Powered by Editorial Manager® and ProduXion Manager® from Aries Systems Corporation

Manuscript (Revision)

Click here to access/download;Manuscript;PLOS_One_smokingandMS_ma

The relationship between smoking cigarettes and metabolic syndrome: a cross-sectional study with non-single residents of Seoul under 40 years old

Sun Woo Kim, MD[1]¶, Ho Jun Kim, MD[b]¶, Kyungha Min, MD[a] , Hobeom Lee, MD[b] , Sung-Ha, Lee, PhD[d] , Sunyoung Kim, MD[c] , Jong Seung Kim, MD[b] , Bumjo Oh, MD[b*]

[a]*Department of Family Medicine, Seoul National University Hospital, Seoul, Republic of Korea*

[b]*Department of Family Medicine, SMG-SNU Boramae Medical Center, Seoul, Republic of Korea*

[c]*Department of Family Medicine, Kyung Hee University Medical Center, Seoul, Republic of Korea*

[d]*Center for Happiness Studies, Seoul National University, Seoul, Republic of Korea*

¶ *These authors contributed equally to this work.*

[*]Send correspondence to: **Bumjo Oh**, MD, MPH.

Department of Family Medicine, Seoul National University Boramae Hospital, Seoul National University College of Medicine, Boramae-ro 5-gil 20, Dongjak-gu, Republic of Korea

E-mail: bumjo.oh@gmail.com

Telephone: 82-2-870-2682

Fax: 82-2-831-0714

Abstract

Introduction: Young adults receive health screenings at lower rates than other age groups, and it may be difficult to detect diseases in the early stages for this group. We examined differences in health status relative to smoking in a young age group using the results of health screenings conducted in engaged and newly married couples in a cross-sectional database.

Methods: The participants in this study were 808 young adults who visited a municipal hospital health screening center from July 2017 to March 2019. They completed a self-administered questionnaire, and physical measurements and a blood test were taken. They were classified into non-cigarette smokers, past cigarette smokers, and current cigarette smokers according to smoking behavior. In this study, we compared metabolic syndrome, the main components of which include obesity, high blood pressure, high blood triglycerides, low levels of HDL cholesterol and insulin resistance, with smoking behavior.

Results: The mean age of the participants was 30.9±3.3 years (males 32.0±3.2, females 29.8±3.1), and 13.9% were current cigarette smokers (males 22.8%, females 5.1%). The proportion of men in their 30s was 76.6% for male group and 50.0% for female group, indicating that the male group had a relatively higher proportion of older and current smokers. Significant differences were found in age, sex, blood pressure, metabolic abnormalities, and drinking status according to smoking status. Cigarette smokers had a 2.4-fold greater risk of metabolic syndrome (95% confidence interval [CI], 1.43–3.96) than non-cigarette smokers; in particular, they had a 2.6-fold (95% CI, 1.44–4.55) greater risk of hypertriglyceridemia and a three-fold (95% CI, 1.45–6.35) greater risk of low HDL cholesterol.

Conclusions: In comparison with non-single, young and generally healthy city dwellers, the risk of metabolic syndrome was significantly higher in smokers than in non-smokers, and in particular, it was confirmed that the risk of hypertriglyceridemia and low HDL cholesterolemia was higher. Smoking cessation is necessary, even for the young, because smoking may cause changes in blood lipids even if the smoking duration is short.

Keywords: non-single residents, Health Screening, cigarette smokers, Metabolic Syndrome

INTRODUCTION

Recent demographic changes that have been occurring in South Korea include falling fertility and an aging population.[1] In particular, one of the noticeable changes that has occurred is the number of marriages and the typical age of first marriage. The annual number of marriages in South Korea increased over a long period, from 295.1 thousand in 1970 to 419.7 thousand in 1992, but decreased slightly afterwards to 332.0 thousand in 2000, 314.3 thousand in 2005, and then 326.1 thousand in 2010[1]. As the number of marriages has been decreasing, the average age at first marriage has been increasing. A survey of the social and economic situation of young people in 2016 reported that 56.0% of respondents said that marriage was necessary, and the average age of their expected marriage was 30.1 years[2]. However, marriage and divorce statistics from the National Statistical Office in 2018 show an average age at first marriage of 33.2 years for men and 30.4 for women and an increase of 0.2 years for both men and women; these values were 1.8 years higher for men and 2.2 years higher for women than their equivalents 10 years ago[3]. The increase in marriage age is complexly connected with problems in education, including the extension of the time necessary to complete higher education, with labor market problems, such as a general lack of positions and lack of good quality ones in particular, with changes in norms and thinking about marriage, and with changes in the marriage market, including the pool of potential male and female partners [1]. Several analyses have indicated that this trend will not change.

Because the population group aged 20–30 years includes the current average age of marriage and is largely healthy, they often avoid proper health assessments. However, as marriage age increases, poor health in members of this age group can cause important health and sociological problems. Subjective health conditions were highest among those in their 20s (35.3%) followed by those in their 30s (32.9%) (National Health and Nutrition Survey, 2017) [4]. As subjective health conditions drop to 20% for those in in their 40s and older, it is highly likely that those in their 20s and 30s do not understand their health as well as other age groups. In particular, young men in their 20s and 30s had health checkup rates of 36.5–55.4%, lower than those for other age groups, making it difficult to diagnose hypertension, diabetes mellitus, or dyslipidemia early[4]. The rate of unmet medical care, which measures necessary

medical services that are left unperformed, was also highest among men in their 30s, at 11.5%, and third highest among men in their 20s. The main reasons for unmet medical care included lack of time, symptoms that are not burdensome, and economic reasons[5]. Smoking rates were the second highest among men in their 30s, at 25.5%, and third highest among men in their 20s, at 24.4%. Monthly drinking rates were the highest among men in their 20s, at 50.6%, and second highest among men in their 30s, at 42.6%[4], implying that men in their 20s and 30s do not take proper care of their health and tend to lead unhealthy lifestyles.

Metabolic syndrome refers to the co-occurrence of abdominal obesity, dyslipidemia, elevated blood pressure, and impaired fasting glucose. When metabolic syndrome is neglected, the risk of cardio-cerebrovascular disease, diabetes mellitus, and cancer increases.[6-8] The prevalence of metabolic syndrome is increasing in South Korea[9], but there have been no independent surveys of metabolic syndrome among persons in their 20s and 30s, who often do not properly assess their health.

In younger age groups, smoking habits may not be seriously taken because the duration of the habit is often not long, and many believe that they will have many chances to quit at a later time. If it can be confirmed that smoking among various lifestyles at a healthy young age is related to a chronic disease state represented by metabolic syndrome, it is expected that this will serve as a basis for recommending smoking cessation to them. Therefore, this study tried to investigate this association by analyzing the health checkup results of engaged and newlywed persons who visited a health examination center at a general hospital in Seoul from July 2017 to March 2019.

METHODS

Study population and data collection

The study population was 808 people who visited the health promotion center of a municipal hospital from July 3, 2017, to March 31, 2019, including of 288 in 2017, 480 in 2018, and 40 in 2019, with no overlap. This population consisted of couples engaged or married less than one year, proved with documents such as wedding invitations, wedding hall use agreements, and resident registration

certificates. The screening items were a self-administered questionnaire, anthropometry, blood tests, urine tests, stool tests, electrocardiograms, pulmonary function tests, image examination, and gastroscopy. Part of the cost of the examination was borne by the subjects, and the remainder was covered by a sponsorship fund and contributions.

Ethics approval and consent to participate

This study was approved by the Institutional Review Board of SMG-SNU Boramae Medical Center (registration number: 30-2018-101). Written informed consent confirming voluntary participation was obtained from the participants. All individually identifying records were anonymized prior to analysis.

Study method

1) Anthropometry and health-related behavior survey

Height and weight were measured with an automatic height–weight measuring instrument (same for all subjects). The subjects were fasting, wearing their examination clothes, and wearing no shoes. The measured height (cm) and weight (kg) values were recorded to one decimal place. The body mass index was calculated as weight (kg)/height (m^2). In the WHO Asian-Pacific criteria, body mass index values smaller than 18.5 kg/m^2 are underweight, 18.5 kg/m^2–23 kg/m^2 are normal weight, 23 kg/m^2–25 kg/ m^2 are overweight, and greater than 25 kg/m^2 are obese[10]. Waist circumferences were measured in the upright posture at the end of normal expiration at the midpoint between the lower border of the rib cage and the top of the iliac crest, following the WHO recommendations. Blood pressure was measured twice using an automatic blood pressure gauge after the subjects had relaxed for at least 10 minutes while they were sitting in a chair, leaning against the back of the chair, their feet placed on the floor without their legs crossed, and with the height of the upper arm adjusted to be equal to the height of the heart.

Health-related behaviors were identified using a self-administered questionnaire on smoking and drinking. The subjects were classified into non-cigarette smokers who had not smoked at least 100

cigarettes in their lifetime and were not currently smoking, previous cigarette smokers who had smoked at least 100 cigarettes in their lifetime but were not currently smoking, and current cigarette smokers who had smoked at least 100 cigarettes in their lifetime and were currently smoking. The kinds of alcoholic beverages drunk were collected. All subjects who reported that they did not drink alcohol at all were considered non-drinkers, and those who wrote that they drank one or more times were considered drinkers.

2) *Blood tests*

Blood tests were conducted on serum separated from blood collected from the brachial vein after a state of fasting for at least 12 hours, and fasting blood glucose, glycated hemoglobin, total cholesterol, triglyceride, high-density lipoprotein (HDL) cholesterol, and low-density lipoprotein (LDL) cholesterol were measured using a Chemistry Analyzer (Roche-Hitachi).

3) *Definition of metabolic syndrome*

The National Cholesterol Education Program/Adult Treatment Panel III defined metabolic syndrome as when at least three of five the following components appear: abdominal obesity, impaired fasting glucose, hypertriglyceridemia, low HDL cholesterolemia, and high blood pressure.[11] Abdominal obesity was assessed using a waist circumference of >90 cm for men and >85 cm for women. Impaired fasting glucose was defined as ≥100 mg/dL or use of an oral hypoglycemic agent or insulin. Hypertriglyceridemia was defined as ≥150 mg/dL or receipt of lipid-lowering drug therapy. Low HDL cholesterolemia was defined as an HDL level of <40 mg/dL for men and <50 mg/dL for women or receipt of lipid-lowering drug therapy. High blood pressure was defined as systolic blood pressure of ≥130 mmHg, or diastolic blood pressure of ≥85 mmHg, or taking a blood pressure medication.

4) *Statistical analysis*

The general characteristics of the study subjects such as age, sex, blood pressure, individual metabolic abnormalities, and drinking status were examined using frequency analyses and compared using chi-square tests among the study subjects, who were classified into non-cigarette smokers, previous cigarette smokers, and current cigarette smokers.[11] In addition, after adjusting for age, sex, drinking, and underlying disease using multivariable logistic regression analyses, the effects of smoking

state on metabolic syndrome and individual metabolic abnormalities were assessed. The survey data were compiled using Microsoft Excel and STATA ver. 15.0 was used for statistical analyses. Significance was assessed at $p < 0.05$.

RESULTS

Characteristics of study subjects

The total number of subjects was 808, and the mean age was 30.88 ± 3.29 years. Among these, 398 were men, and 410 were women. Among the subjects, 72.52% were non-cigarette smokers, 13.61% were previous cigarette smokers, and 13.86% were current cigarette smokers; 67.20% were current drinkers. Metabolic syndrome was found in 17.45% of subjects; in 31.91% of men and 4.63% of women. The ratio of those with underlying diseases was only 2.60% of all subjects (Table 1).

Table 1. Descriptive characteristics of study participants

Variables	Total	Men, N (%)	Women, N (%)
**Number of people**			
Proportion, N (%)	808	398 (49.26)	410 (50.74)
**Age, years**			
Mean (±SD)	30.88 (3.29)	31.99(3.15)	29.80 (3.06)
20–29 years old, N (%)	298 (36.88)	93 (23.37)	205 (50.00)
30–39 years old, N (%)	510 (63.12)	305 (76.63)	205 (50.00)
**Blood pressure**			
Mean SBP (±SD)	113.64 (13.43)	121.05 (12.60)	106.45(9.83)
Mean DBP (±SD)	77.96 (9.80)	81.91 (10.07)	74.13 (7.82)
Normal, N (%)	614 (75.99)	245 (61.56)	369 (90.00)
Increasing Blood Pressure, N (%)	194 (24.01)	153 (38.44)	41 (10.00)
**Fasting blood glucose**			
Mean (±SD)	86.93 (10.70)	89.95 (12.09)	84.01 (8.16)
Normal, N (%)	759 (93.94)	353 (88.69)	406 (99.02)
Increasing Fasting blood glucose, N (%)	49 (6.06)	45 (11.31)	4 (0.98)
**Abdominal obesity**[a]			
Mean (± SD)	81.04 (10.78)	88.33 (8.75)	73.98 (7.31)
Normal weight, N (%)	613 (75.87)	241 (60.55)	372 (90.73)
Obesity, N (%)	195 (24.13)	157 (39.45)	38 (9.27)
**General obesity**[b]			

Mean (±SD)	22.75 (3.61)	24.89 (3.27)	20.68 (2.56)
Normal weight, N (%)	612 (75.74)	221 (55.53)	391 (95.37)
Obesity, N (%)	196 (24.26)	177 (44.47)	19 (4.63)
**Metabolic syndrome[c]**			
Normal, N (%)	667 (82.55)	271 (68.09)	396 (96.59)
Metabolic syndrome, N (%)	141 (17.45)	127 (31.91)	14 (3.41)
**Smoking**			
Non-cigarette smoker	586 (72.52)	212 (53.27)	374 (91.22)
Past cigarette smoker	110 (13.61)	95 (23.87)	15 (3.66)
Current cigarette smoker	112 (13.86)	91 (22.86)	21 (5.12)
**Alcohol use**			
Non-drinker	262 (32.43)	96 (24.12)	166 (40.49)
Drinker	543 (67.20)	300 (75.38)	243 (59.27)
**Presence of comorbidity[d]**			
No	787 (97.40)	380 (95.48)	407 (99.27)
Yes	21 (2.60)	18 (4.52)	3 (0.73)

Values are presented as count number (weighted %).

[a]Abdominal obesity (Korean criteria): waist circumference 90 cm in men and ≥ 85 cm in women.

[b]General obesity (Korean criteria): body mass index (BMI) ≥ 25 kg m2.

[c]Metabolic syndrome (Korean criteria): person has three or more of the following measurements: 1. Abdominal obesity (waist circumference 90 cm in men, 85 in women), 2. Triglyceride level ≥ 150 mg/dL, 3. HDL cholesterol < 40 mg/dL in men or < 50 mg/dL in women or on dyslipidemia medication, 4. Systolic blood pressure (top number) ≥ 130 mm Hg or diastolic blood pressure (bottom number) ≥ 85 mmHg or on hypertension medication, 5. Fasting glucose ≥ 100 mg/dL or on diabetes medication

[d]Comorbidity related to metabolic syndrome

Comparison of characteristics of study subjects according to smoking status

Table 2 provides a comparison of general characteristics, such as age, sex, blood pressure, individual metabolic abnormalities, and drinking status among smoking status groups, which showed significant differences in all characteristics except underlying diseases (Table 2).

Table 2. Comparison of subject characteristics according to smoking status

Variables	Non-cigarette smoker	Past cigarette smoker	Current cigarette smoker	P-value[a]
**Number of people** N (%)	586 (72.52)	110 (13.61)	112 (13.86)	
**Age, years**				
20–29 years	246 (41.98)	29 (26.36)	23 (20.54)	<0.005
30–39 years	340 (58.02)	81 (73.64)	89 (79.46)	
**Sex**				<0.005

Men	212 (36.18)	95 (86.36)	91 (81.25)	
Women	374 (63.82)	15 (13.64)	21 (18.75)	
**Blood pressure**				
Normal, N (%)	473 (80.72)	70 (63.64)	71 (63.39)	<0.005
Increasing blood pressure, N (%)	113 (19.28)	40 (36.36)	41 (36.61)	
**Fasting blood glucose**				
Normal	560 (95.56)	100 (90.91)	99 (88.39)	0.005
Increasing fasting blood glucose	26 (4.44)	10 (9.09)	13 (11.61)	
**Triglyceride**				
Normal	541 (92.32)	91 (82.73)	75 (66.96)	<0.005
Increasing triglycerides	45 (7.68)	19 (17.27)	37 (33.04)	
**High Density Lipoprotein**				
Normal	550 (93.86)	99 (90.00)	94 (83.93)	<0.005
Decreasing HDL	36 (6.14)	11 (10.00)	18 (16.07)	
**Alcohol use**				
Non-drinker	223 (38.05)	18 (16.36)	21 (18.75)	<0.005
Drinker	361 (61.60)	92 (83.64)	90 (80.36)	
**Presence of comorbidity**[d]				
No	575 (98.12)	106 (96.36)	106 (94.64)	0.08
Yes	11 (1.88)	4 (3.64)	6 (5.36)	
**Abdominal obesity**[a]				
Normal weight	477 (81.40)	69 (62.73)	67 (59.82)	<0.005
Obesity	109 (18.60)	41 (37.27)	45 (40.18)	
**General obesity**[b]				
Normal weight	480 (81.91)	66 (60.00)	66 (58.93)	<0.005
Obesity	106 (18.09)	44 (40.00)	46 (41.07)	
**Metabolic syndrome**[c]				
Normal	667 (82.55)	271 (68.09)	396 (96.59)	<0.005
Metabolic syndrome	141 (17.45)	127 (31.91)	14 (3.41)	

[a]Analyzed by chi-square test.

[a]Abdominal obesity (Korean criteria): waist circumference 90 cm in men and ≥ 85 cm in women.

[b]General obesity (Korean criteria): body mass index (BMI) ≥ 25 kg m2.

[c]Metabolic syndrome (Korean criteria): person has three or more of the following measurements: 1. Abdominal obesity (waist circumference 90 cm in men, 85 in women), 2. Triglyceride level ≥ 150 mg/dL, 3. HDL cholesterol < 40 mg/dL in men or < 50 mg/dL in women or on dyslipidemia medication, 4. Systolic blood pressure (top number) ≥ 130 mm Hg or diastolic blood pressure (bottom number) ≥ 85 mmHg or on hypertension medication, 5. Fasting glucose ≥ 100 mg/dL or on diabetes medication

[d]Comorbidity related to metabolic syndrome

Prevalence rates of metabolic syndrome and individual metabolic abnormalities according to smoking status

Table 3 shows the associations between smoking, obesity, metabolic syndrome, and individual components of metabolic syndrome after covariate adjustment. Cigarette smokers had a 2.38 times higher risk for metabolic syndrome than non-cigarette smokers, 2.56 times higher risk for hypertriglyceridemia, and 3.03 times higher risk for low HDL cholesterolemia.

Table 3. Crude and adjusted odds ratios (and 95% confidence intervals) from logistic regression analyses identifying associations between smoking status and components of metabolic syndrome

Components	Non-smoker	Past smoker	Current smoker
Proportion, N (%)	586 (72.52)	110 (13.61)	112 (13.86)
General obesity[a]	106 (18.09)	44 (40.00)	46 (41.07)
Crude OR (95% CI)	1	**3.02** (1.95–4.67)	**3.22** (2.10–4.95)
Adjusted OR[b] (95% CI)	1	1.10 (0.68–1.79)	1.23 (0.75–2.01)
Metabolic syndrome[c]	30 (5.12)	15 (13.64)	21 (18.75)
Crude OR (95% CI)	1	**2.20** (1.32–3.67)	**5.34** (3.40–8.37)
Adjusted OR[d] (95% CI)	1	0.88 (0.50–1.53)	**2.38** (1.43–3.96)
Abdominal obesity[e]	109 (18.60)	41 (37.27)	45 (40.18)
Crude OR (95% CI)	1	**2.60** (1.68–4.03)	**2.90** (1.88–4.45)
Adjusted OR[f] (95% CI)	1	1.24 (0.76–2.01)	1.43 (0.89–2.31)
Increased blood pressure	113 (19.28)	40 (36.36)	41 (36.61)
Crude OR (95% CI)	1	**2.39** (1.54–3.71)	**2.42** (1.56–3.74)
Adjusted OR[g] (95% CI)	1	1.13 (0.68–1.88)	0.94 (0.56–1.59)
Increased fasting blood glucose	26 (4.44)	10 (9.09)	13 (11.61)
Crude OR (95% CI)	1	**2.15** (1.00–4.60)	**2.83** (1.41–5.69)
Adjusted OR[h] (95% CI)	1	0.94 (0.42–2.12)	1.03 (0.47–2.25)
Increased triglycerides	45 (7.68)	19 (17.27)	37 (33.04)
Crude OR (95% CI)	1	**2.51** (1.40–4.48)	**5.93** (3.61–9.75)
Adjusted OR[i] (95% CI)	1	1.04 (0.55–1.98)	**2.56** (1.44–4.55)
Decreased high-density lipoprotein	36 (6.14)	11 (10.00)	18 (16.07)
Crude OR (95% CI)	1	1.70 (0.84–3.45)	**2.93** (1.59–5.37)
Adjusted OR[j] (95% CI)	1	2.26 (0.98–5.20)	**3.03** (1.45–6.35)

OR, odds ratio; CI, confidence intervals. The numbers in bold indicate statistically significant differences in ($p < .05$) odds for smoking status in a given group as compared to the reference (non-smoker) group.

[a]General obesity (Korean criteria): body mass index (BMI) ≥ 25 kg m^2.

[b]Adjusted for age, sex, drinking, comorbidity

[c]Metabolic syndrome (Korean criteria): person has three or more of the following measurements:

1. Abdominal obesity (waist circumference 90 cm in men, 85 in women), 2. Triglyceride level ≥ 150 mg/dL, 3. HDL cholesterol < 40 mg/dL in men or < 50 mg/dL in women or on dyslipidemia

medication, 4. Systolic blood pressure (top number) ≥ 130 mm Hg or diastolic blood pressure (bottom number) ≥ 85 mmHg or on hypertension medication, 5. Fasting glucose ≥ 100 mg/dL or on diabetes medication

[d]Adjusted for age, sex, drinking, comorbidity

[e]Abdominal obesity (Korean criteria): waist circumference >90 cm in men, >85 in women

[f]Adjusted for age, sex, drinking, comorbidity.

[g]Adjusted for age, sex, drinking, comorbidity, waist circumference, triglycerides, high density lipoprotein, Fasting blood glucose

[h]Adjusted for age, sex, drinking, comorbidity, waist circumference, triglycerides, high density lipoprotein, blood pressure.

[i]Adjusted for age, sex, drinking, comorbidity, waist circumference, high density lipoprotein, blood pressure, Fasting blood glucose

[j]Adjusted for age, sex, drinking, comorbidity, waist circumference, triglycerides, blood pressure, Fasting blood glucose

DISCUSSION

In this study, the authors found that smoking was significantly associated with metabolic syndrome in urban living and healthy young populations. Compared to nonsmokers, smokers had a 2.4 times higher risk of metabolic syndrome. Moreover, the current findings support that smoking increases blood triglyceride levels and lowers HDL cholesterol levels, indicating that the changes in blood lipid levels caused by smoking may play an important role in the association between smoking and metabolic syndrome[12].

Smoking induces an increase in body insulin-antagonistic hormones, such as cortisol, catecholamine, and growth hormone, and it induces increases in lipolysis.[13] A previous finding indicated that this increased triglyceride levels[14]. In addition, nicotine levels in the body promote fat degradation. The free fatty acids increased through these pathways damage the beta cells of the pancreas, which is thought to cause impaired fasting glucose[15]. It was found in the literature that by synthesizing these mechanisms, metabolic syndrome-related factors are significantly identified in diabetic smokers.[16]

In young adults in their 20s and 30s, the duration or amount of smoking is likely to be relatively low compared to older age groups, but among those who were cigarette smokers, the risk of dyslipidemia was significantly higher than among non-cigarette smokers. In addition, although this was

not statistically significant, previous cigarette smokers had a higher risk of dyslipidemia than non-cigarette smokers. Combining the previous findings, we can infer that current or past smoking is associated with dyslipidemia, separate from the period of smoking. In order to confirm this, an additional comparison can be made by classifying light or heavy smokers according to the amount and duration of smoking, but we divided them into three groups: current smokers, non-smokers, and former smokers. This was because the definition of light smoker was not clear and the number of subjects was small, so if the category was further divided by the new definition, statistical power could be reduced. However, considering that there is a possibility of a dose-response relationship between the amount of smoking and metabolic syndrome, it was thought that large-scale data analysis would be necessary regarding this point in the future.

The risk of metabolic syndrome was statistically significantly higher among cigarette smokers than non-cigarette smokers. Previous studies indicate that smoking is significantly associated with metabolic syndrome[17-19]. Moreover, among the components of metabolic syndrome, abdominal obesity, elevated blood pressure, and impaired fasting glucose were found to have a higher prevalence in the smoking group[18]. In this study, cigarette smokers and previous cigarette smokers showed a higher risk for abdominal obesity and elevated blood pressure than non-cigarette smokers, although the differences were not statistically significant. Given the results obtained with the 20-30 age group, who are the subjects of this study, and the results of previous studies, it is thought that smoking increases the risk of metabolic syndrome regardless of age at the beginning of smoking or the duration of smoking. However, compared to the non-smoker group, the previous cigarette smokers group was not significantly different not only in metabolic syndrome and its components, but also in general obesity. This suggests that quitting smoking at a young age may lower the risk of metabolic syndrome compared to cigarette smokers.

Previous studies report that more cigarette smokers have poor lifestyle habits, including drinking and inactivity, in addition to smoking, and these habits increase the risk of metabolic disease[20]. In particular, it is well known that alcohol consumption is a major cause of metabolic syndrome. However, a possible limitation of this study is that when the questionnaires were collected, the authors were unable

to accurately determine the type and amount of alcohol that subjects drank. For the convenience of the research, a drinker is defined as “drinking more than once a week,” so the proportion of drinkers is high. Therefore, we acknowledge that more meaningful results could have been confirmed if the subjects were classified into moderate/risk drinking according to the amount of alcohol consumed and the relationship with metabolic syndrome risk factors had been further analyzed. Although this study’s findings cannot be compared with previous work because the amount of drinking and activity was not evaluated, this tendency to have poor habits is believed to appear in this age group. This study suggests that even relatively young age groups should pay attention to their lifestyle habits. After the above findings were combined, it was seen that smoking cessation could be an important improvement in lifestyle habits that lowers the prevalence of dyslipidemia and metabolic syndrome.

The cross-sectional studies we conducted have the following general limitations. First, because it was a cross-sectional study, estimating the cause-effect relationship between smoking and metabolic syndrome was complicated. Second, this study cannot be used to represent the entire population in this age group because it was conducted only among those who sought a health checkup at a municipal hospital within a specific period of time. Third, because the amount of drinking was measured in a self-administered questionnaire, it was challenging to evaluate drinking accurately. This may have produced errors during statistical analysis.

Nevertheless, to the best of the our knowledge, this may be a very rare study of smoking and metabolic syndrome in a young, healthy adult population under 40 years of age. Other studies using health screening data have mainly concentrated on older age groups, and no study has been conducted with relatively young age groups. A significant strength of this study is its identification of significant results, although the subjects were young adults with short smoking durations. It is suggested that subsequent studies with more sophisticated designs be performed to overcome some of the aforementioned limitations. In addition, based on the current results, it is expected that this will serve as a basis for recommending quitting smoking among young people with short smoking periods and no other diseases.

Declaration of Interests

No potential conflict of interest relevant to this article is reported.

Funding

This work was not supported by private or public funders.

REFERENCES

1. Kim TH, Kim DS, Kim EJ, Bae HJ, Min HJ. A Study on The Effect of Late Marriage on Low Fertility in Korea. Korea Institute for Health and Social Affairs, 2011.
2. Kim KH, Ha HS, Shin IC. Survey on the actual social and economic conditions of the youth. National Youth Policy Institute, 2016.
3. Marriage and Divorce Statistics in 2018. Statistics Korea, 2019.
4. Oh B, Han D-H, Han K-T, Liu X, Ukken J, Chang C, et al. Association between residual teeth number in later life and incidence of dementia: A systematic review and meta-analysis. BMC Geriatrics. 2018;18(1):48. doi: 10.1186/s12877-018-0729-z.
5. Kim C-G, Lee SH, Cha SK. Influencing Factors on Cardio-cerebrovascular Disease Risk Factors in Young Men: Focusing on Obesity Indices. Journal of Korean Biological Nursing Science. 2017;19(1):1-10. doi: 10.7586/jkbns.2017.19.1.1.
6. Detection NCEPEPo, Adults ToHBCi. Third report of the National Cholesterol Education Program (NCEP) expert panel on detection, evaluation, and treatment of high blood cholesterol in adults (adult treatment panel III): National Cholesterol Education Program, National Heart, Lung, and Blood ...; 2002.
7. Lemieux I, Pascot As, Couillard C, Lamarche Bt, Tchernof A, Alméras N, et al. Hypertriglyceridemic waist: a marker of the atherogenic metabolic triad (hyperinsulinemia; hyperapolipoprotein B; small, dense LDL) in men? Circulation. 2000;102(2):179-84.
8. Esposito K, Chiodini P, Colao A, Lenzi A, Giugliano D. Metabolic syndrome and risk of cancer: a systematic review and meta-analysis. Diabetes care. 2012;35(11):2402-11.
9. Tran BT, Jeong BY, Oh J-K. The prevalence trend of metabolic syndrome and its components and risk factors in Korean adults: results from the Korean National Health and Nutrition Examination Survey 2008–2013. BMC Public Health. 2017;17(1):71.
10. Organization WH. The Asia-Pacific perspective: redefining obesity and its treatment: Sydney: Health Communications Australia; 2000.
11. Chen C-C, Li T-C, Chang P-C, Liu C-S, Lin W-Y, Wu M-T, et al. Association among cigarette smoking, metabolic syndrome, and its individual components: the metabolic syndrome study in Taiwan. Metabolism. 2008;57(4):544-8.
12. Craig WY, Palomaki GE, Haddow JE. Cigarette smoking and serum lipid and lipoprotein concentrations: an analysis of published data. Bmj. 1989;298(6676):784-8.
13. Andersson K, Arner P. Systemic nicotine stimulates human adipose tissue lipolysis through local cholinergic and catecholaminergic receptors. International journal of obesity. 2001;25(8):1225-32.
14. Cena H, Fonte ML, Turconi G. Relationship between smoking and metabolic syndrome. Nutrition reviews. 2011;69(12):745-53.
15. Bergman RN, Ader M. Free fatty acids and pathogenesis of type 2 diabetes mellitus. Trends in Endocrinology & Metabolism. 2000;11(9):351-6.

16. Kar D, Gillies C, Zaccardi F, Webb D, Seidu S, Tesfaye S, et al. Relationship of cardiometabolic parameters in non-smokers, current smokers, and quitters in diabetes: a systematic review and meta-analysis. Cardiovasc Diabetol. 2016;15(1):158. PubMed PMID: 27881170.
17. Geslain-Biquez C, Vol S, Tichet J, Caradec A, D'hour A, Balkau B, et al. The metabolic syndrome in smokers. The DESIR study. Diabetes & metabolism. 2003;29(3):226-34.
18. Oh SW, Yoon YS, Lee ES, Kim WK, Park C, Lee S, et al. Association between cigarette smoking and metabolic syndrome: the Korea National Health and Nutrition Examination Survey. Diabetes care. 2005;28(8):2064-6.
19. Hong AR, Lee KS, Lee SY, Yu JH. Association of current and past smoking with metabolic syndrome in men. J Prev Med Public Health. 2009;42(3):160-4.
20. Oh JE. Association between Smoking Status and Metabolic Syndrome in Men. Korean Journal of Obesity. 2014;23(2).

DATA SET

Click here to access/download
Supporting Information
mw_lab_171819_01_mod.dta

The relationship between smoking cigarettes and metabolic syndrome: a cross-sectional study with non-single residents of Seoul under 40 years old

Formatted: Highlight

Commented [B1]: 1)Title – I am not quite sure why the authors have chosen 'marriage' as a determinant of metabolic syndrome? Marriage may not happen, and people can live together for decades. It does not affect their risk of developing metabolic syndrome.

Answer: We agree with the reviewer's opinion. This was a kind of business, and the purpose was to recommend the health care of newlyweds, and I think that the title of this manuscript, written for research purposes, needs to be revised. So, we have changed it to:

Sun Woo Kim, MD,[a†] Ho Jun Kim, MD,[b†] Kyungha Min, MD,[a] Hobeom Lee, MD,[b] Sung-Ha, Lee, PhD,[d] Sunyoung Kim, MD,[c] Jong Seung Kim, MD,[b] Bumjo Oh, MD[b*]

[a]*Department of Family Medicine, Seoul National University Hospital, Seoul, Republic of Korea,* [b]*Department of Family Medicine, SMG-SNU Boramae Medical Center, Seoul, Republic of Korea,* [c]*Department of Family Medicine, Kyung Hee University Medical Center, Seoul, Republic of Korea,* [d]*Center for Happiness Studies, Seoul National University, Seoul, Republic of Korea*

† These authors contributed equally to this work.

[*]~~Correspondence~~ Send correspondence to: Bumjo Oh, MD, MPH.
Department of Family Medicine, Seoul National University Boramae Hospital, Seoul National University College of Medicine, Boramae-ro 5-gil 20, Dongjak-gu [07061]

E-mail: bumjo.oh@gmail.com
Telephone: 82-2-870-2682.
Fax: 82-2-831-0714

Abstract

Introduction: Young adults receive health screenings at lower rates than other age groups, and it may be difficult to detect diseases in the~~ir~~ early stages for this group. We examine<u>d</u> differences in health status relative to smoking in a young age group~~,~~ using the results of health screenings conducted in engaged and newly married couples in a cross-sectional database.

Methods: <u>The participants in this study were</u> ~~Eight hundred eight~~<u>808</u> young adults who visited a municipal hospital health screening center from July 2017 to March 2019 ~~participated in this study~~. They completed a self-administered questionnaire, <u>and</u> physical measurements~~,~~ and a blood test <u>were taken</u>. They classified into non-cigarette smokers, past cigarette smokers, and current cigarette smokers according to smoking behavior. ~~The~~ <u>In this study, we compared</u> ~~main components of~~ metabolic syndrome<u>, the main components of which</u> include obesity, high blood pressure, high blood triglycerides, low levels of HDL cholesterol and insulin resistance<u>, with smoking behavior</u>.

Results: The mean age of the participants was 30.9±3.3 years (male<u>s</u> 32.0±3.2, female<u>s</u> 29.8±3.1), and 13.9% ~~of them~~ were current cigarette smokers (male<u>s</u> 22.8%, female<u>s</u> 5.1%). In the male group, 76.6% were in their ~~thirties~~<u>30s</u>, but 50.0% were female, so the male group had a relatively higher proportion of ~~age~~ <u>older</u> and current smokers. Significant differences were found in age, sex, blood pressure, metabolic abnormalities, and drinking status according to smoking status. Cigarette smokers had a 2.4-fold greater risk of metabolic syndrome (95% confidence interval [CI], 1.43–3.96) than non-cigarette smokers; in particular, they had a 2.6-fold (95% CI, 1.44–4.55) greater risk of hypertriglyceridemia and a ~~3~~<u>three</u>-fold (95% CI, 1.45–6.35) greater risk of low HDL cholesterol.

Conclusions: In comparison with non-single, young and generally healthy city dwellers, the risk of metabolic syndrome was significantly higher in smokers than in non-smokers, and in particular, it was confirmed that the risk of hypertriglyceridemia and low HDL cholesterolemia was higher. Smoking cessation is necessary, even for the young, because smoking may cause changes in blood lipids even if the smoking duration is short.

Commented [B2]: 1)*Results* – Please add standard deviation of age and male female split. This is going to help the reader to understand dispersion of data. Male female split will be helpful as you have mentioned the uptake of screening tests are comparatively lower in young males.
<u>Answer:</u> Thank you for your comment. Men and women were separated and SD was indicated.

Commented [53]: This is unclear ("In the male group…50.0% were female")

Keywords: non-single residents, Health Screening, cigarette smokers, Metabolic Syndrome

Formatted: Highlight

INTRODUCTION

~~The r~~Recent demographic changes that have been occurring in South Korea~~,~~ ~~including~~ include falling fertility and an aging population.[1] ~~Especially~~In particular, one of the noticeable changes that has ~~occurs~~ occurred ~~in~~ is the number of marriages and the typical age of first marriage. The annual number of marriages in South Korea increased over a long period, from 295.1 thousand in 1970 to 419.7 thousand in 1992, but decreased slightly afterwards to 332.0 thousand in 2000, 314.3 thousand in 2005, and then 326.1 thousand in 2010[1]. As the number of marriages has been decreasing, the average age at first marriage has been increasing. A survey ~~(~~of the social and economic situation~~)~~ of young people in 2016 reported that 56.0% of respondents ~~stated~~ said that marriage was necessary, and the average age of their expected marriage was 30.1 years[2]. However, ~~according the~~ marriage and divorce statistics from the National Statistical Office in 2018 show an average age at first marriage of 33.2 years for men and 30.4 for women and an increase of 0.2 years for both men and women; these values were 1.8 years higher for men and 2.2 years higher for women than their equivalents ~~ten~~ 10 years ago[3]. The ~~problem of~~ increase~~s~~ in marriage age is complexly connected with problems in education, including the extension of the time necessary to complete higher education, with labor market problems, such as ~~the~~a general lack of positions ~~generally~~ and ~~the~~ lack of good quality ones in particular, with changes in norms and thinking about marriage, and with changes in the marriage market, including the pool of potential male and female partners ~~men and women partner pools~~[1]. Several analyses have indicated that this trend will not change.

Because the population group aged 20–30 years includes the current average age of marriage and is largely healthy, they often avoid proper health assessment~~s of their health~~. However, as marriage age increases, poor health in members of this age group can cause important health and sociological problems. Subjective health conditions were highest among those in their 20s (35.3%) followed by those in their 30s (32.9%) (National Health and Nutrition Survey, 2017) [4]. As subjective health conditions drop~~ped~~ to 20% for those in in their 40s and older ~~groups~~, it is highly likely that those ~~persons~~ in their 20s and 30s do not understand their health as well as other age groups. In particular, young men in their 20s and 30s had health checkup rates of 36.5–55.4%, lower than those for other age groups,

Formatted: Highlight

making it difficult to diagnose ~~determine~~ hypertension, diabetes mellitus, or dyslipidemia early[4]. The rate of unmet medical care, which measures necessary medical services that ~~were~~ are left unperformed, ~~were~~ was also highest among men in their 30s, at 11.5%, and third highest among men in their 20s. The main reasons for unmet medical care included lack of time, symptoms that are not burdensome, and economic reasons[5]. Smoking rates were the second~~-~~ highest among men in their 30s, at 25.5%, and third highest among men in their 20s, at 24.4%. Monthly drinking rates were the highest among men in their 20s, at 50.6%, and second~~-~~ highest among men in their 30s, at 42.6%[4], implying that men in their 20s and 30s do not take proper care ~~for~~ of their health and tend to lead unhealthy lifestyles.

Metabolic syndrome refers to the co-occurrence ~~is the composite occurrence~~ of abdominal obesity, dyslipidemia, elevated blood pressure, and impaired fasting glucose. When ~~the~~ metabolic syndrome is neglected, the risk ~~for~~ of cardio-cerebrovascular disease, diabetes mellitus, and cancer increases.[6-8] The prevalence of ~~the~~ metabolic syndrome is increasing in South Korea[9], but there ~~has~~ have been no independent surveys of ~~the~~ metabolic syndrome among persons in their 20s and 30s, who often do not properly assess their health.

In younger age groups, smoking habits may not be seriously taken because the duration of the habit is often not long, and many believe that they will have many chances to quit at a later time. If it can be confirmed that smoking among various lifestyles ~~in~~ at a healthy young age is related to a chronic disease state represented by metabolic syndrome, it is expected that this will serve as a basis for recommending smoking cessation to them. Therefore, this study tried to investigate this association by analyzing the health checkup results of engaged and newlywed~~s~~ persons who visited a health examination center at a general hospital in Seoul from July 2017 to March 2019.

Formatted: Highlight

Commented [B4]: 1)"Therefore, this study is intended to conduct cross-sectional studies based on the results of health screenings of engaged and newly married couples who visited the health examination center of a general hospital in Seoul from July 2017 to March 2019 in order to analyze differences in the health status, various clinical tests, and prevalence between smoking and non-smoking among *existing living habits* in relatively young age groups" – too long sentence not sure what the authors are trying to say. Please rewrite the sentence.
Answer: We agree with the reviewer's opinion, and have corrected the sentence as pointed out.

METHODS

Study population and data collection

The study population was ~~selected from~~ 808 people who visited the health promotion center of a municipal hospital from July 3, 2017, to March 31, 2019, including of 288 in 2017, 480 in 2018, and

40 in 2019, with no overlap. The This population consists consisted of couples engaged or married less than one year ago, proved with certificates documents such as wedding invitations, wedding hall use agreements, and resident registration certificates. The screening items were a self-administered questionnaire, anthropometry, blood tests, urine tests, stool tests, electrocardiograms, pulmonary function tests, image examination, and gastroscopy. Part of the cost of the examination was borne by the subjects, and the remainder was covered by a sponsorship fund and contributions.

Ethics approval and consent to participate

This study was approved by the institutional Institutional review Review board Board of SMG-SNU Boramae Medical Center (registration number: 30-2018-101). Written informed consent confirming voluntary participation was obtained from the participants. All individually identifying records were anonymized prior to analysis.

Study method

1) Anthropometry and health-related behavior survey

Height and weight were measured with an automatic height–weight measuring instrument (same for all subjects). The subjects were fasting, wearing their examination clothes, and wearing no shoes. The measured height (cm) and weight (kg) values were recorded to one decimal place. The body mass index was calculated as weight (kg)/height (m^2). In the WHO Asian-Pacific criteria, body mass index values smaller than 18.5 kg/m^2 is are underweight, 18.5 kg/m^2–23 kg/m^2 is are normal weight, 23 kg/m^2–25 kg/ m^2 is are overweight, and greater than 25 kg/m^2 is are obese[10]. Waist circumferences were measured in the upright posture at the end of normal expiration at the midpoint between the lower border of the rib cage and the top of the iliac crest, following the WHO recommendations. Blood pressure was measured twice using an automatic blood pressure gauge after the subjects had relaxed for at least 10 minutes while they were sitting in a chair, leaning against the back of the chair, their feet placed on the floor without having their legs crossed, and with the height of the upper arm adjusted to be equal to the height of the heart.

Health-related behaviors were identified using a self-administered questionnaire on smoking and drinking. The subjects were classified into non-cigarette smokers who had not smoked at least 100 cigarettes in their lifetime and were not currently smoking, previous cigarette smokers who had smoked at least 100 cigarettes in their lifetime but were not currently smoking, and current cigarette smokers who had smoked at least 100 cigarettes in their lifetime and were currently smoking. The kinds of alcoholic beverages drunk were collected. All subjects who reported that they did not drink alcohol at all were considered non-drinkers, and those who wrote that they drank one or more times were considered drinkers.

2) Blood tests

Blood tests were conducted on serum separated from blood collected from the brachial vein after a state of fasting for at least 12 hours~~ was identified~~, and fasting blood glucose, glycated hemoglobin, total cholesterol, triglyceride, high-density lipoprotein (HDL) cholesterol, and low-density lipoprotein (LDL) cholesterol were measured using a Chemistry ~~Auto a~~Analyzer (Roche-Hitachi).

3) Definition of metabolic syndrome

The National Cholesterol Education Program/Adult Treatment Panel III defined metabolic syndrome as when at least three of five the following components appear: abdominal obesity, impaired fasting glucose, hypertriglyceridemia, low HDL cholesterolemia, and high blood pressure.[11]~~.~~ Abdominal obesity was assessed using a waist circumference of >90 cm for men and >85 cm ~~in~~ for women. Impaired fasting glucose was defined as ≥100 mg/dL or use of an oral hypoglycemic agent or insulin. Hypertriglyceridemia was defined as ≥150 mg/dL or receipt of lipid-lowering drug therapy. Low HDL cholesterolemia was defined as an HDL level of <40 mg/dL for men and <50 mg/dL for women or receipt of lipid-lowering drug therapy. High blood pressure was defined as systolic blood pressure of ≥130 mmHg, or diastolic blood pressure of ≥85 mmHg, or taking a blood pressure medication.

4) Statistical analysis

The general characteristics of the study subjects such as age, sex, blood pressure, individual metabolic abnormalities, and drinking status were examined using frequency analyses and compared

using chi-square tests among the study subjects, who were classified into non-cigarette smokers, previous cigarette smokers, and current cigarette smokers.[11] In addition, after adjusting for age, sex, drinking, and underlying disease using multivariable logistic regression analyses, the effects of smoking state on metabolic syndrome and individual metabolic abnormalities were assessed. The survey data were compiled using Microsoft Excel and STATA ver. 15.0 was used for statistical analyses. Significance was assessed at $p < 0.05$.

RESULTS

Characteristics of study subjects

The total number of subjects was 808, and the mean age was 30.88 ± 3.29 years. Among these, 398 were men, and 410 were women. Among the subjects, 72.52% were non-cigarette smokers, 13.61% were previous cigarette smokers, and 13.86% were current cigarette smokers; 67.20% were current drinkers. Metabolic syndrome was found in 17.45% of subjects~~,~~; in 31.91% of men~~,~~ and 4.63% of women. The ratio of those with underlying diseases was only 2.60% of all subjects (Table 1).

Table 1. Descriptive characteristics of study participants

Variables	Total	Men, N (%)	Women, N (%)
**Number of people**			
Proportion, N (%)	808	398 (49.26)	410 (50.74)
**Age, years**			
Mean (±SD)	30.88 (3.29)	31.99(3.15)	29.80 (3.06)
20–29 years old, N (%)	298 (36.88)	93 (23.37)	205 (50.00)
30–39 years old, N (%)	510 (63.12)	305 (76.63)	205 (50.00)
**Blood pressure**			
Mean SBP (±SD)	113.64 (13.43)	121.05 (12.60)	106.45(9.83)
Mean DBP (±SD)	77.96 (9.80)	81.91 (10.07)	74.13 (7.82)
Normal, N (%)	614 (75.99)	245 (61.56)	369 (90.00)
Increasing Blood Pressure, N (%)	194 (24.01)	153 (38.44)	41 (10.00)
**Fasting ~~Blood~~ blood ~~Glucose~~glucose**			
Mean (±SD)	86.93 (10.70)	89.95 (12.09)	84.01 (8.16)
Normal, N (%)	759 (93.94)	353 (88.69)	406 (99.02)

Increasing Fasting blood glucose, N (%)	49 (6.06)	45 (11.31)	4 (0.98)
**Abdominal ~~Obesity~~[a] obesity[a]**			
Mean (± SD)	81.04 (10.78)	88.33 (8.75)	73.98 (7.31)
Normal weight, N (%)	613 (75.87)	241 (60.55)	372 (90.73)
Obesity, N (%)	195 (24.13)	157 (39.45)	38 (9.27)
**General ~~Obesity~~[b] obesity[b]**			
Mean (±SD)	22.75 (3.61)	24.89 (3.27)	20.68 (2.56)
Normal weight, N (%)	612 (75.74)	221 (55.53)	391 (95.37)
Obesity, N (%)	196 (24.26)	177 (44.47)	19 (4.63)
**Metabolic syndrome[c]**			
Normal, N (%)	667 (82.55)	271 (68.09)	396 (96.59)
Metabolic syndrome, N (%)	141 (17.45)	127 (31.91)	14 (3.41)
**Smoking**			
Non-cigarette~~s~~ smoker	586 (72.52)	212 (53.27)	374 (91.22)
Past cigarette~~s~~ smoker	110 (13.61)	95 (23.87)	15 (3.66)
Current cigarette~~s~~ ~~Smoker~~smoker	112 (13.86)	91 (22.86)	21 (5.12)
**Alcohol use**			
Non-drinker	262 (32.43)	96 (24.12)	166 (40.49)
Drinker	543 (67.20)	300 (75.38)	243 (59.27)
**Presence of comorbidity[d]**			
No	787 (97.40)	380 (95.48)	407 (99.27)
Yes	21 (2.60)	18 (4.52)	3 (0.73)

Values are presented as count number (weighted %).

[a]Abdominal obesity (Korean criteria): waist circumference 90 cm in men and ≥ 85 cm in women.

[b]General obesity (Korean criteria): body mass index (BMI) ≥ 25 kg m2.

[c]Metabolic syndrome (Korean criteria): person has three or more of the following measurements:

1. Abdominal obesity (waist circumference 90 cm in men, 85 in women), 2. Triglyceride level ≥ 150 mg/dL, 3. HDL cholesterol < 40 mg/dL in men or < 50 mg/dL in women or on dyslipidemia medication, 4. Systolic blood pressure (top number) ≥ 130 mm Hg or diastolic blood pressure (bottom number) ≥ 85 mmHg or on ~~Hypertension~~ hypertension medication, 5. Fasting glucose ≥ 100 mg/dL or on diabetes medication

[d]Comorbidity related to metabolic syndrome

Comparison of characteristics of study subjects according to smoking status

Table 2 provides a comparison of general characteristics, such as age, sex, blood pressure, individual metabolic abnormalities, and drinking status among smoking ~~state~~ status groups, which showed significant differences in all characteristics~~,~~ except underlying diseases (Table 2).

Table 2. Comparison of subject characteristics according to smoking status

Variables	Non-cigarettes smoker	Past cigarettes smoker	Current cigarettes smoker	P-value[a]
**Number of people** N (%)	586 (72.52)	110 (13.61)	112 (13.86)	
**Age, years**				
20–29 years	246 (41.98)	29 (26.36)	23 (20.54)	<0.005
30–39 years	340 (58.02)	81 (73.64)	89 (79.46)	
**Sex**				
Men	212 (36.18)	95 (86.36)	91 (81.25)	<0.005
Women	374 (63.82)	15 (13.64)	21 (18.75)	
**Blood pressure**				
Normal, N (%)	473 (80.72)	70 (63.64)	71 (63.39)	<0.005
Increasing ~~Blood~~ blood ~~Pressure~~pressure, N (%)	113 (19.28)	40 (36.36)	41 (36.61)	
**Fasting ~~Blood~~ blood ~~Glucose~~glucose**				
Normal	560 (95.56)	100 (90.91)	99 (88.39)	0.005
Increasing ~~Fasting~~ fasting blood glucose	26 (4.44)	10 (9.09)	13 (11.61)	
**Triglyceride**				
Normal	541 (92.32)	91 (82.73)	75 (66.96)	<0.005
Increasing ~~Triglyceride~~triglycerides	45 (7.68)	19 (17.27)	37 (33.04)	
**High Density Lipoprotein**				
Normal	550 (93.86)	99 (90.00)	94 (83.93)	<0.005
Decreasing HDL	36 (6.14)	11 (10.00)	18 (16.07)	
**Alcohol use**				
Non-drinker	223 (38.05)	18 (16.36)	21 (18.75)	<0.005
Drinker	361 (61.60)	92 (83.64)	90 (80.36)	
**Presence of comorbidity**[d]				
No	575 (98.12)	106 (96.36)	106 (94.64)	0.08
Yes	11 (1.88)	4 (3.64)	6 (5.36)	
**Abdominal Obesity**[a]				
Normal weight	477 (81.40)	69 (62.73)	67 (59.82)	<0.005
Obesity	109 (18.60)	41 (37.27)	45 (40.18)	
**General Obesity**[b]				
Normal weight	480 (81.91)	66 (60.00)	66 (58.93)	<0.005
Obesity	106 (18.09)	44 (40.00)	46 (41.07)	
**Metabolic syndrome**[c]				
Normal	667 (82.55)	271 (68.09)	396 (96.59)	<0.005
Metabolic syndrome	141 (17.45)	127 (31.91)	14 (3.41)	

[a]Analyzed by chi-square test.

[a]Abdominal obesity (Korean criteria): waist circumference 90 cm in men and ≥ 85 cm in women.
[b]General obesity (Korean criteria): body mass index (BMI) ≥ 25 kg m2.
[c]Metabolic syndrome (Korean criteria): person has three or more of the following measurements:
1. Abdominal obesity (waist circumference 90 cm in men, 85 in women), 2. Triglyceride level ≥ 150 mg/dL, 3. HDL cholesterol < 40 mg/dL in men or < 50 mg/dL in women or on dyslipidemia medication, 4. Systolic blood pressure (top number) ≥ 130 mm Hg or diastolic blood pressure (bottom number) ≥ 85 mmHg or on ~~Hypertension~~ hypertension medication, 5. Fasting glucose ≥ 100 mg/dL or on diabetes medication
[d]Comorbidity related to metabolic syndrome

Prevalence rates of metabolic syndrome and individual metabolic abnormalities according to smoking status

Table 3 shows the associations between smoking, obesity, metabolic syndrome, and individual components of metabolic syndrome after covariate adjustment. Cigarette smokers had a 2.38 times ~~the~~ higher risk for metabolic syndrome than non-cigarette smokers, 2.56 times higher ~~the~~ risk for hypertriglyceridemia, and 3.03 times higher ~~the~~ risk for low HDL cholesterolemia.

Table 3. Crude and adjusted odds ratios (and 95% confidence intervals) from logistic regression analyses identifying associations between smoking status and components of metabolic syndrome

Components	Non-smoker	Past smoker	Current smoker
Proportion, N (%)	586 (72.52)	110 (13.61)	112 (13.86)
General obesity[a]	106 (18.09)	44 (40.00)	46 (41.07)
Crude OR (95% CI)	1	**3.02** (1.95–4.67)	**3.22** (2.10–4.95)
Adjusted OR[b] (95% CI)	1	1.10 (0.68–1.79)	1.23 (0.75–2.01)
Metabolic syndrome[c]	30 (5.12)	15 (13.64)	21 (18.75)
Crude OR (95% CI)	1	**2.20** (1.32–3.67)	**5.34** (3.40–8.37)
Adjusted OR[d] (95% CI)	1	0.88 (0.50–1.53)	**2.38** (1.43–3.96)
Abdominal obesity[e]	109 (18.60)	41 (37.27)	45 (40.18)
Crude OR (95% CI)	1	**2.60** (1.68–4.03)	**2.90** (1.88–4.45)
Adjusted OR[f] (95% CI)	1	1.24 (0.76–2.01)	1.43 (0.89–2.31)
Increased ~~Blood~~ blood pressure	113 (19.28)	40 (36.36)	41 (36.61)
Crude OR (95% CI)	1	**2.39** (1.54–3.71)	**2.42** (1.56–3.74)
Adjusted OR[g] (95% CI)	1	1.13 (0.68–1.88)	0.94 (0.56–1.59)
Increased ~~Fasting~~ fasting blood glucose	26 (4.44)	10 (9.09)	13 (11.61)
Crude OR (95% CI)	1	**2.15** (1.00–4.60)	**2.83** (1.41–5.69)
Adjusted OR[h] (95% CI)	1	0.94 (0.42–2.12)	1.03 (0.47–2.25)
Increased ~~Triglycerides~~triglycerides	45 (7.68)	19 (17.27)	37 (33.04)
Crude OR (95% CI)	1	**2.51** (1.40–4.48)	**5.93** (3.61–9.75)

Adjusted OR[i] (95% CI)	1	1.04 (0.55–1.98)	**2.56** (1.44–4.55)
Decreased ~~High~~ high-density lipoprotein	36 (6.14)	11 (10.00)	18 (16.07)
Crude OR (95% CI)	1	1.70 (0.84–3.45)	**2.93** (1.59–5.37)
Adjusted OR[j] (95% CI)	1	2.26 (0.98–5.20)	**3.03** (1.45–6.35)

OR, odds ratio; CI, confidence intervals. ~~Boldface~~ The numbers in bold indicate~~s~~ statistically significant differences in ($p < .05$) odds for smoking status in ~~the~~ a given group~~,~~ as compared to the reference (non-smoker) group.

[a]General obesity (Korean criteria): body mass index (BMI) ≥ 25 kg m².

[b]Adjusted for age, sex, drinking, comorbidity

[c]Metabolic syndrome (Korean criteria): person has three or more of the following measurements:

1. Abdominal obesity (waist circumference 90 cm in men, 85 in women), 2. Triglyceride level ≥ 150 mg/dL, 3. HDL cholesterol < 40 mg/dL in men or < 50 mg/dL in women or on dyslipidemia medication, 4. Systolic blood pressure (top number) ≥ 130 mm Hg or diastolic blood pressure (bottom number) ≥ 85 mmHg or on ~~Hypertension~~ hypertension medication, 5. Fasting glucose ≥ 100 mg/dL or on diabetes medication

[d]Adjusted for age, sex, drinking, comorbidity

[e]Abdominal obesity (Korean criteria): waist circumference >90 cm in men, >85 in women

[f]Adjusted for age, sex, drinking, comorbidity.

[g]Adjusted for age, sex, drinking, comorbidity, waist circumference, triglycerides, high density lipoprotein, Fasting blood glucose

[h]Adjusted for age, sex, drinking, comorbidity, waist circumference, triglycerides, high density lipoprotein, blood pressure.

[i]Adjusted for age, sex, drinking, comorbidity, waist circumference, high density lipoprotein, blood pressure, Fasting blood glucose

[j]Adjusted for age, sex, drinking, comorbidity, waist circumference, triglycerides, blood pressure, Fasting blood glucose

Commented [B5]: 1)Table 3 – Can the author clarify what is meant by crude OR and adjusted OR, please?
Answer: As you have pointed out, we added a description in Table 3.

DISCUSSION

~~Through~~ In this study, ~~we~~ the authors found that smoking was significantly associated with metabolic syndrome in urban living and healthy young populations. Compared to nonsmokers, smokers had a 2.4 times higher risk of metabolic syndrome. Moreover, the current findings support~~s~~ that smoking increases blood triglyceride levels and lowers HDL cholesterol levels, indicating that the changes in blood lipid levels caused by smoking may play an important role in the association between smoking and metabolic syndrome[12].

Commented [B6]: 1)Why the authors decided only to comment of lipid profiles rather than the whole spectrum of components of metabolic syndrome?
Answer: As mentioned in the Conclusion, this study confirmed that the prevalence of metabolic syndrome was higher in the current smoker group than in the non-smoker group. In particular, it was confirmed that the adjusted OR significantly increased in TG and HDL-C (high-density lipoprotein) among metabolic components. In this regard, the discussion was focused on the relevant risk factors. The authors emphasize this intention by amending the first sentence of the first paragraph of the Discussion.

Smoking induces an increase in body insulin-antagonistic hormones, such as cortisol, catecholamine, and growth hormone, and it induces increases in lipolysis.[13] A previous finding

indicated that this increased triglyceride levels[14]. In addition, nicotine levels in the body promote fat degradation. The free fatty acids increased through these pathways damage the beta cells of the pancreas, which is thought to cause impaired fasting glucose[15]. It was found in the literature that ~~By~~ by synthesizing these mechanisms, ~~the literature that~~ metabolic syndrome-related factors are significantly identified in diabetic smokers ~~can also be found~~.[16]

In young adults in their 20s and 30s, the duration or amount of smoking is likely to be relatively low compared to older age groups, but among those ~~that~~ who were cigarette smokers, the risk of dyslipidemia was significantly higher than among non-cigarette smokers. In addition, although this was not statistically significant, previous cigarette smokers had a higher risk of dyslipidemia than non-cigarette smokers. Combining the previous findings, we can infer that current or past smoking is associated with dyslipidemia, separate from the period of smoking. In order to confirm this, an additional comparison can be made by classifying light or heavy smokers according to the amount and duration of smoking, but we divided them into three groups: current smokers, non-smokers, and former smokers. This was because the definition of light smoker was not clear and the number of subjects was small, so if the category was further divided by the new definition, statistical power could be reduced. However, considering that there is a possibility of a dose-response relationship between the amount of smoking and ~~the~~ metabolic syndrome, it was thought that ~~a~~ large-scale data analysis would be necessary ~~considering~~ regarding this point in the future.

The risk of metabolic syndrome was statistically significantly higher among cigarette smokers than non-cigarette smokers. Previous studies indicate that smoking is significantly associated with metabolic syndrome[17-19]. Moreover, ~~(~~among the components of metabolic syndrome,~~)~~ abdominal obesity, elevated blood pressure, and impaired fasting glucose ~~have been~~were found to have a higher prevalence in the smoking group[18]. In this study, cigarette smokers and previous cigarette smokers showed a higher risk for abdominal obesity and elevated blood pressure than non-cigarette smokers, although the differences were not statistically significant. Given the results obtained with the 20-30 age group~~s~~, who are the subjects of this study, and the results of previous studies, it is thought that smoking increases the risk of metabolic syndrome regardless of ~~the~~ age ~~of~~ at the beginning of smoking or the duration of

Commented [B7]: 2)Relationship between smoking and the components of metabolic syndrome is well established - https://pubmed.ncbi.nlm.nih.gov/27881170/
Answer: Thank you for your comment. We cited and mentioned it in the last sentence of the second paragraph of the Discussion after reviewing the contents of the review article.

smoking. However, compared to the non-smoker group, the previous cigarette smokers group was not significantly different not only in metabolic syndrome and its components, but also in general obesity compared to the non-smoker group. This suggests that quitting smoking at a young ages may lower the risk of metabolic syndrome compared to cigarette smokers.

Previous studies report that more cigarette smokers have poor lifestyle habits, including drinking and inactivity, in addition to smoking, and these habits increase the risk of metabolic disease[20]. In particular, it is well known that alcohol consumption is a major cause of metabolic syndrome. However, it may be a possible limitation of this study is that when the questionnaires were collected, the authors were unable to accurately determine the type and amount of alcohol that subjects drank when collecting the questionnaire. For the convenience of the researcher, a drinker is defined as '"drinking more than once a week' is defined as a drinker," so the proportion of drinkers is high. Therefore, we acknowledge that more meaningful results could have been confirmed if the subjects were classified into moderate/risk drinking according to the amount of alcohol consumed and the relationship with metabolic syndrome risk factors had been further analyzed. Although this study's findings cannot be compared with previous work because the amount of drinking and activity was not evaluated, this tendency to have poor habits is believed to appear in this age group. This study suggests that even relatively young age groups should pay attention to their lifestyle habits. After the above findings were combined, it was seen that smoking cessation could be an important improvement in lifestyle habits that lowers the prevalence of dyslipidemia and metabolic syndrome.

The cross-sectional studies we conducted generally have the following general limitations. First, because it was a cross-sectional study, estimating the cause-effect relationship between smoking and metabolic syndrome is was complicated. Second, this study cannot be used to represent the entire population in this age group because it was conducted only among those who sought a health checkup at a municipal hospital within a specific period of time. Third, because the amount of drinking was measured in a self-administered questionnaire, it was challenging to evaluate drinking accurately. This may have produced errors during statistical analysis.

Nevertheless, to the best of the author's our knowledge, this may be a very rare study of smoking

and metabolic syndrome in a young, healthy adult population under 40 years of age. Other studies using health screening data have mainly concentrated ~~mainly~~ on older age groups, and no study has been conducted with relatively young age groups. A significant strength of this study is its identification of significant results, although the subjects were young adults with short smoking durations. It is suggested that subsequent studies ~~of~~ with more sophisticated designs be performed to overcome some of the aforementioned limitations. In addition, based on the current results, it is expected that this will serve as a basis for recommending quitting smoking among young people with short smoking periods and no other diseases.

Commented [B8]: 3)The authors could inform the reader how this relationship is different in young people in their 20s and 30s.

Answer: Thank you for your comment. The aforementioned papers are included as references. In addition, compared with previous studies, a significant increase in TG and a significant decrease in HDL were characteristic in smokers who were younger subjects under the age of 40, and this was mentioned in the Discussion section

Declaration of Interests

No potential conflict of interest relevant to this article is reported.

Funding

This work was not supported by ~~no~~ private or public funders~~.~~.

REFERENCES

1. Kim TH, Kim DS, Kim EJ, Bae HJ, Min HJ. A Study on The Effect of Late Marriage on Low Fertility in Korea. Korea Institute for Health and Social Affairs, 2011.
2. Kim KH, Ha HS, Shin IC. Survey on the actual social and economic conditions of the youth. National Youth Policy Institute, 2016.
3. Marriage and Divorce Statistics in 2018. Statistics Korea, 2019.
4. Oh B, Han D-H, Han K-T, Liu X, Ukken J, Chang C, et al. Association between residual teeth number in later life and incidence of dementia: A systematic review and meta-analysis. BMC Geriatrics. 2018;18(1):48. doi: 10.1186/s12877-018-0729-z.
5. Kim C-G, Lee SH, Cha SK. Influencing Factors on Cardio-cerebrovascular Disease Risk Factors in Young Men: Focusing on Obesity Indices. Journal of Korean Biological Nursing Science. 2017;19(1):1-10. doi: 10.7586/jkbns.2017.19.1.1.
6. Detection NCEPEPo, Adults ToHBCi. Third report of the National Cholesterol Education Program (NCEP) expert panel on detection, evaluation, and treatment of high blood cholesterol in adults (adult treatment panel III): National Cholesterol Education Program, National Heart, Lung, and Blood ...; 2002.
7. Lemieux I, Pascot As, Couillard C, Lamarche Bt, Tchernof A, Alméras N, et al. Hypertriglyceridemic waist: a marker of the atherogenic metabolic triad (hyperinsulinemia; hyperapolipoprotein B; small, dense LDL) in men? Circulation. 2000;102(2):179-84.
8. Esposito K, Chiodini P, Colao A, Lenzi A, Giugliano D. Metabolic syndrome and risk of cancer: a systematic review and meta-analysis. Diabetes care. 2012;35(11):2402-11.
9. Tran BT, Jeong BY, Oh J-K. The prevalence trend of metabolic syndrome and its components and risk factors in Korean adults: results from the Korean National Health and Nutrition Examination Survey 2008–2013. BMC Public Health. 2017;17(1):71.
10. Organization WH. The Asia-Pacific perspective: redefining obesity and its treatment: Sydney: Health Communications Australia; 2000.
11. Chen C-C, Li T-C, Chang P-C, Liu C-S, Lin W-Y, Wu M-T, et al. Association among cigarette smoking, metabolic syndrome, and its individual components: the metabolic syndrome study in Taiwan. Metabolism. 2008;57(4):544-8.
12. Craig WY, Palomaki GE, Haddow JE. Cigarette smoking and serum lipid and lipoprotein concentrations: an analysis of published data. Bmj. 1989;298(6676):784-8.
13. Andersson K, Arner P. Systemic nicotine stimulates human adipose tissue lipolysis through local cholinergic and catecholaminergic receptors. International journal of obesity. 2001;25(8):1225-32.
14. Cena H, Fonte ML, Turconi G. Relationship between smoking and metabolic syndrome. Nutrition reviews. 2011;69(12):745-53.
15. Bergman RN, Ader M. Free fatty acids and pathogenesis of type 2 diabetes mellitus. Trends in Endocrinology & Metabolism. 2000;11(9):351-6.

16. Kar D, Gillies C, Zaccardi F, Webb D, Seidu S, Tesfaye S, et al. Relationship of cardiometabolic parameters in non-smokers, current smokers, and quitters in diabetes: a systematic review and meta-analysis. Cardiovasc Diabetol. 2016;15(1):158. PubMed PMID: 27881170.
17. Geslain-Biquez C, Vol S, Tichet J, Caradec A, D'hour A, Balkau B, et al. The metabolic syndrome in smokers. The DESIR study. Diabetes & metabolism. 2003;29(3):226-34.
18. Oh SW, Yoon YS, Lee ES, Kim WK, Park C, Lee S, et al. Association between cigarette smoking and metabolic syndrome: the Korea National Health and Nutrition Examination Survey. Diabetes care. 2005;28(8):2064-6.
19. Hong AR, Lee KS, Lee SY, Yu JH. Association of current and past smoking with metabolic syndrome in men. J Prev Med Public Health. 2009;42(3):160-4.
20. Oh JE. Association between Smoking Status and Metabolic Syndrome in Men. Korean Journal of Obesity. 2014;23(2).

Response to Editor

PONE-D-20-15624R1

Relationship between Smoking and Metabolic Syndrome in Engaged and Newly Married Couples Adults in Their 20s and 30s

Dear Editor,

We like to thank you and the reviewers for the very helpful advices.
Please find our revision and answers to the open points enclosed.

Best regards,
Bumjo Oh

ABSTRACT

1) Title – I am not quite sure why the authors have chosen 'marriage' as a determinant of metabolic syndrome? Marriage may not happen, and people can live together for decades. It does not affect their risk of developing metabolic syndrome.

Answer: We agree with the reviewer's opinion. This was a kind of business, and the purpose was to recommend the health care of newlyweds, and I think that the title of this manuscript, written for research purposes, needs to be revised. So, we have changed it to: **The relationship between smoking cigarettes and metabolic syndrome: a cross-sectional study with non-single residents of Seoul under 40 years old**

2) *Metabolic syndrome was defined using Adult Treatment Panel III*.- Please tell the reader what is meant by the adult treatment panel.

Answer: Thank you for your comment. The content was explained by quoting from the methodology, not the abstract.

3) *Results* – Please add standard deviation of age and male female split. This is going to help the reader to understand dispersion of data. Male female split will be helpful as you have mentioned the uptake of screening tests are comparatively lower in young males.

Answer: Thank you for your comment. Men and women were separated and SD was indicated.

4) Conclusion – Glycosylated Haemoglobin, smoking and metabolic syndrome are closely linked. Why the authors only commented on TG and HDL and not other components of the metabolic syndrome?

Answer: In this study, we looked at the relationship between smoking and metabolic syndrome, and further looked at the relationship between the components that define metabolic syndrome. Through this, the Conclusion emphasized and mentioned this part with the result that TG and HDL had a significant relationship with smoking.

INTRODUCTION

1) The annual number of marriages in South Korea showed increases over a long period – correct grammar.

Answer: We have corrected the sentence as you pointed out.

2) "Metabolic syndrome is the composite occurrence of abdominal obesity, dyslipidemia, elevated blood pressure, and impaired fasting glucose" – what about BMI and waist circumference?

Answer: Thank you for your comment. The relationship between obesity index and metabolic syndrome risk factors has been suggested in various ways, and the authors selected one of them according to the criteria of Adult Treatment Panel III. This is because BMI and waist circumference are both used as indicators of obesity, but if they are selected at the same time, covariance may play a role.

3) "Therefore, this study is intended to conduct cross-sectional studies based on the results of health screenings of engaged and newly married couples who visited the health examination center of a general hospital in Seoul from July 2017 to March 2019 in order to analyze differences in the health status, various clinical tests, and prevalence between smoking and non-smoking among *existing living habits* in relatively young age groups" – too long sentence not sure what the authors are trying to say. Please rewrite the sentence.

Answer: We agree with the reviewer's opinion, and have corrected the sentence as pointed out.

STUDY METHOD

1) Statistical analysis – "In addition, after *compensating* for age, sex, drinking, ……" adjusting would be a better word than compensating.

Answer: We agree with the reviewer's opinion, and changed the word as you instructed.

2) Table 3 – Can the author clarify what is meant by crude OR and adjusted OR, please?

Answer: As you have pointed out, we added a description in Table 3.

DISCUSSION

1) Why the authors decided only to comment of lipid profiles rather than the whole spectrum of components of metabolic syndrome?

Answer: As mentioned in the Conclusion, this study confirmed that the prevalence of metabolic syndrome was higher in the current smoker group than in the non-smoker group. In particular, it was confirmed that the adjusted OR significantly increased in TG and HDL-C (high-density lipoprotein) among metabolic components. In this regard, the discussion was focused on the relevant risk factors. The authors emphasize this intention by amending the first sentence of the first paragraph of the Discussion.

2) Relationship between smoking and the components of metabolic syndrome is well established - https://pubmed.ncbi.nlm.nih.gov/27881170/

Answer: Thank you for your comment. We cited and mentioned it in the last sentence of the second paragraph of the Discussion after reviewing the contents of the review article.

3) The authors could inform the reader how this relationship is different in young people in their 20s and 30s.

Answer: Thank you for your comment. The aforementioned papers are included as references. In addition, compared with previous studies, a significant increase in TG and a significant decrease in HDL were characteristic in smokers who were younger subjects under the age of 40, and this was mentioned in the Discussion section.

5. Review Comments to the Author

Please use the space provided to explain your answers to the questions above. You may also include additional comments for the author, including concerns about dual publication,

research ethics, or publication ethics. (Please upload your review as an attachment if it exceeds 20,000 characters)

Reviewer #1: This is an important area of research to explore how smoking affects the cardiometabolic parameters in people in their 20s and 30s. This paper can be published with some minor corrections. The paper needs overall editing for grammar and punctuation.
Answer: Thank you for your comment. We have completed language editing once again for the revised manuscript.

Reviewer #2: Obesity and smoking are important causes of morbidity and mortality worldwide. Smoking reduces insulin sensitivity, induces insulin resistance, and enhances cardiovascular risk factors, such as elevated plasma triglycerides, reduced high-density lipoprotein–cholesterol, and hyperglycemia. Smoking is associated with metabolic abnormalities and increases the risk of metabolic syndrome.

In this study, the authors evaluated the relationship between smoking and metabolic syndrome in 808 young adults and found that the odds ratio versus nonsmokers for metabolic syndrome, hypertriglyceridemia, and low HDL cholesterolemia was significantly higher in smokers. This study provides a positive association between smoking and metabolic syndrome in young adults.

Some questions need to be addressed. Whether and how drinking affects metabolic syndrome in young adults either in the smoking group or non-smoking group. Was any difference of metabolic syndrome analyzed in the light smokers and heavy smokers, compared to the non-smoking group?

Answer: Thank you for your comments, and we would like to respond to them.
First, the researchers divided the subjects into three groups, namely current smokers, non-smokers, and ex-smokers, according to the definition of ever-smoker, and analyzed them.

1) Since the number of subjects is not large, if the categories are further divided by the new definition, the statistical power may decrease.

2) The definition of light/heavy smoker is not clear.

3) The smoking rate of female subjects is very low.

For these reasons, the authors judged that it would be difficult to identify a significant difference between groups when additional conditions were added for analysis. However, we thought that there is a possibility of a dose-response between the amount of smoking and metabolic syndrome, so this was additionally described in the Discussion section.
Second, we are well aware that alcohol consumption is also a major contributor to metabolic syndrome. However, it was later realized that the authors did not accurately determine the type and amount of alcohol the subjects drank when collecting the questionnaire. For convenience, since “if you drink more than once a week” is defined as a drinker, it is acknowledged that the proportion of drinkers is high. Therefore, the authors agreed that a more meaningful result would have been possible if the relationship with metabolic components was further checked by dividing it into appropriate/risk drinking according to the amount of alcohol consumed. Reflecting these details, an existing reference was added and this content was additionally described as a limitation in the Discussion section.
