# Supplementary material for: The relationship between smoking cigarettes and metabolic syndrome: A cross-sectional study with non-single residents of Seoul under 40 years old
Source: PLoS One. 2021 Aug 19;16(8):e0256257. doi: 10.1371/journal.pone.0256257 (PMC8376018; doi:10.1371/journal.pone.0256257)
Supplement: Attachment — Submitted filename: PLOS One - April 2021.docx [file pone.0256257.s002.docx]

Peer-review PLOS-One

**ABSTRACT**

1) Title – I am not quite sure why the authors have chosen ‘marriage’ as a determinant of metabolic syndrome? Marriage may not happen, and people can live together for decades. It does not affect their risk of developing metabolic syndrome.
2) *Metabolic syndrome was defined using Adult Treatment Panel III*.- Please tell the reader what is meant by the adult treatment panel.
3) *Results* – Please add standard deviation of age and male female split. This is going to help the reader to understand dispersion of data. Male female split will be helpful as you have mentioned the uptake of screening tests are comparatively lower in young males.
4) Conclusion – Glycosylated Haemoglobin, smoking and metabolic syndrome are closely linked. Why the authors only commented on TG and HDL and not other components of the metabolic syndrome?

**INTRODUCTION**

1) The annual number of marriages in South Korea showed increases over a long period – correct grammar.
2) “Metabolic syndrome is the composite occurrence of abdominal obesity, dyslipidemia, elevated blood pressure, and impaired fasting glucose” – what about BMI and waist circumference?
3) “Therefore, this study is intended to conduct cross-sectional studies based on the results of health screenings of engaged and newly married couples who visited the health examination center of a general hospital in Seoul from July 2017 to March 2019 in order to analyze differences in the health status, various clinical tests, and prevalence between smoking and non-smoking among *existing living habits* in relatively young age groups” – too long sentence not sure what the authors are trying to say. Please rewrite the sentence.

STUDY METHOD

1) Statistical analysis – “In addition, after *compensating* for age, sex, drinking, ……” adjusting would be a better word than compensating.
2) Table 3 – Can the author clarify what is meant by crude OR and adjusted OR, please?

DISCUSSION

1) Why the authors decided only to comment of lipid profiles rather than the whole spectrum of components of metabolic syndrome?
2) Relationship between smoking and the components of metabolic syndrome is well established - <https://pubmed.ncbi.nlm.nih.gov/27881170/>
3) The authors could inform the reader how this relationship is different in young people in their 20s and 30s.
